# Post procedure headache in patients treated for neurovascular arteriovenous malformations and aneurysms using endovascular therapy

**DOI:** 10.1186/s10194-016-0666-1

**Published:** 2016-08-22

**Authors:** Sabrina Khan, Faisal Mohammad Amin, John Hauerberg, Markus Holtmannspötter, Julie Falkenberg Petersen, Zainab Fakhril-Din, David Gaist, Messoud Ashina

**Affiliations:** 1 Danish Headache Center, Department of Neurology, Rigshospitalet Glostrup, Faculty of Health and Medical Sciences, University of Copenhagen, Nordre Ringvej 57, Glostrup, 2600 Denmark; 2 Department of Neurosurgery, Rigshospitalet, Faculty of Health and Medical Sciences, University of Copenhagen, Copenhagen, Denmark; 3 Department of Radiology, Rigshospitalet, Faculty of Health and Medical Sciences, University of Copenhagen, Copenhagen, Denmark; 4 Department of Neurology, Odense University Hospital, Odense, Denmark; 5 Department of Clinical Research, Faculty of Health Sciences, University of Southern Denmark, Odense, Denmark

**Keywords:** Endovascular therapy, Arteriovenous malformation, Aneurysm, Headache, Post procedure, Risk

## Abstract

**Background:**

Though endovascular therapy (EVT) is increasingly applied in the treatment of intracranial vascular lesions, little is known about the effect of EVT on post-procedure headache. We aimed to investigate the prevalence of headache in patients who have undergone EVT for cerebral arteriovenous malformations (AVMs) and aneurysms.

**Methods:**

A total of 324 patients underwent EVT treatment for aneurysms and AVMs at the Danish National Hospital from January 2012 to December 2014. We applied strict exclusion criteria in order to minimize the effect of other factors on headache occurrence, e.g., craniotomy. Eligible subjects were phone-interviewed using a purpose-developed semi-structured questionnaire. Headaches were classified according to ICHD-III beta criteria.

**Results:**

The 59 patients underwent treatment of aneurysms (*n* = 43), cranial dural fistulas (*n* = 11), and AVMs (*n* = 5). There was a significant increase in overall headache (*p* = 0.017) and tension-type headache (TTH) (*p* = 0.012) within the first 3 months after EVT compared to 1 month before EVT. However, at interview time (median 2.5 years post-EVT), the increase in overall headache, migraine, and tension-type headache was not statistically significant. A minority of patients experienced headaches for the first time within 3 months of their EVT (migraine 4, TTH 10). At interview time, 50 % of these new headaches still persisted.

**Conclusion:**

Our results suggest a temporary increase in headache in the first 3 months after EVT, which normalizes over time. Clinicians may use this knowledge to better inform their patients of functional outcomes after their EVT procedure.

## Background

Endovascular treatment (EVT) of intracranial vascular lesions as aneurysms or arteriovenous malformations (AVMs) has been increasingly applied in the last decades. Evidence suggests a reduction of post-treatment mortality and morbidity with the transition from open surgery to EVT [1]; however, functional outcomes such as headache after EVT have received only little attention. These vascular lesions may predispose to headache in their own right [2, 3], and several studies describe headache as a presenting symptom of both conditions [4, 5]. What is less known, is how this headache is affected by endovascular treatment procedures, and the expected time frame of headache resolution. Current studies in this field are contradictory, some showing headache improvement after EVT, others worsening of preexisting headaches [6–9]. Furthermore, it is unknown whether the treatment itself may induce headache in a patient without previous headache.

From a clinical perspective, uncovering any risk of post treatment headache and the temporal evolution of such a headache is important for optimal counseling of patients with treatment demanding vascular lesions.

The aim of the present study was to investigate the prevalence of headache in patients with intracranial aneurysms and AVMs after EVT, and to uncover whether EVT treatment predisposes to onset of new headache. For this purpose we retrospectively assessed headache characteristics in a cohort of patients with AVMs and aneurysms who had undergone EVT at a single institution.

## Methods

### Patients and data collection

Using a computerized registry maintained by the Department of Radiology, we identified all patients who underwent EVT for cerebral AVMs and aneurysms at a tertiary referral center at Rigshospitalet (the Danish National Hospital), Copenhagen, Denmark between January 2012 and December 2014. The first author assessed the medical records of all identified cases and based on this information, excluded those fulfilling one or more of the following exclusion criteria: 1) EVT undergone for conditions other than AVM and aneurysms, 2) non-EVT treatment of vessel malformation, e.g., open surgery, 3) multiple EVT treatments, 4) multiple records for the same patient, 5) other intracranial illnesses, 6) head trauma resulting in unconsciousness and hospitalization, 7) aphasia, or no command of Danish or English, 8) aged below 18 years at time of EVT, and 9) unreachable after 5 efforts.

The remaining eligible patients were interviewed via telephone using a purpose-developed semi-structured questionnaire aimed at eliciting any history of headache as well as possible headache after EVT. Headache status was established for the following time periods: (i) 1 year before EVT; (ii) 1 month before EVT; (iii) 3 months after EVT; and (iv) at time of interview. The semi-structured interview allowed for diagnosis of headaches according to the International Classification of Headache Disorders 3 beta version. Headaches where classified as migraine (with and without aura), tension-type headache, and “overall headache”. Overall headache included migraine, tension-type headache, probable migraine, probable tension-type headache, cluster headache, and headaches that could not be classified due to lack of information. The first author and two medical students trained by the first author conducted all interviews in the period of August 2014 to February 2016.

The Committee on Health Research Ethics, Capital Region approved the study. All patients provided written informed consent.

### Statistical analysis

Frequencies and percentages were calculated for categorical variables and medians and range were calculated for continuous variables. For comparisons of categorical data we used McNemars test. All p-values were two-sided and we considered p-values below 0.05 to be statistically significant. All analyses were performed using IBM**®** SPSS**®** Statistics version 23.

## Results

**Fig. 1:**
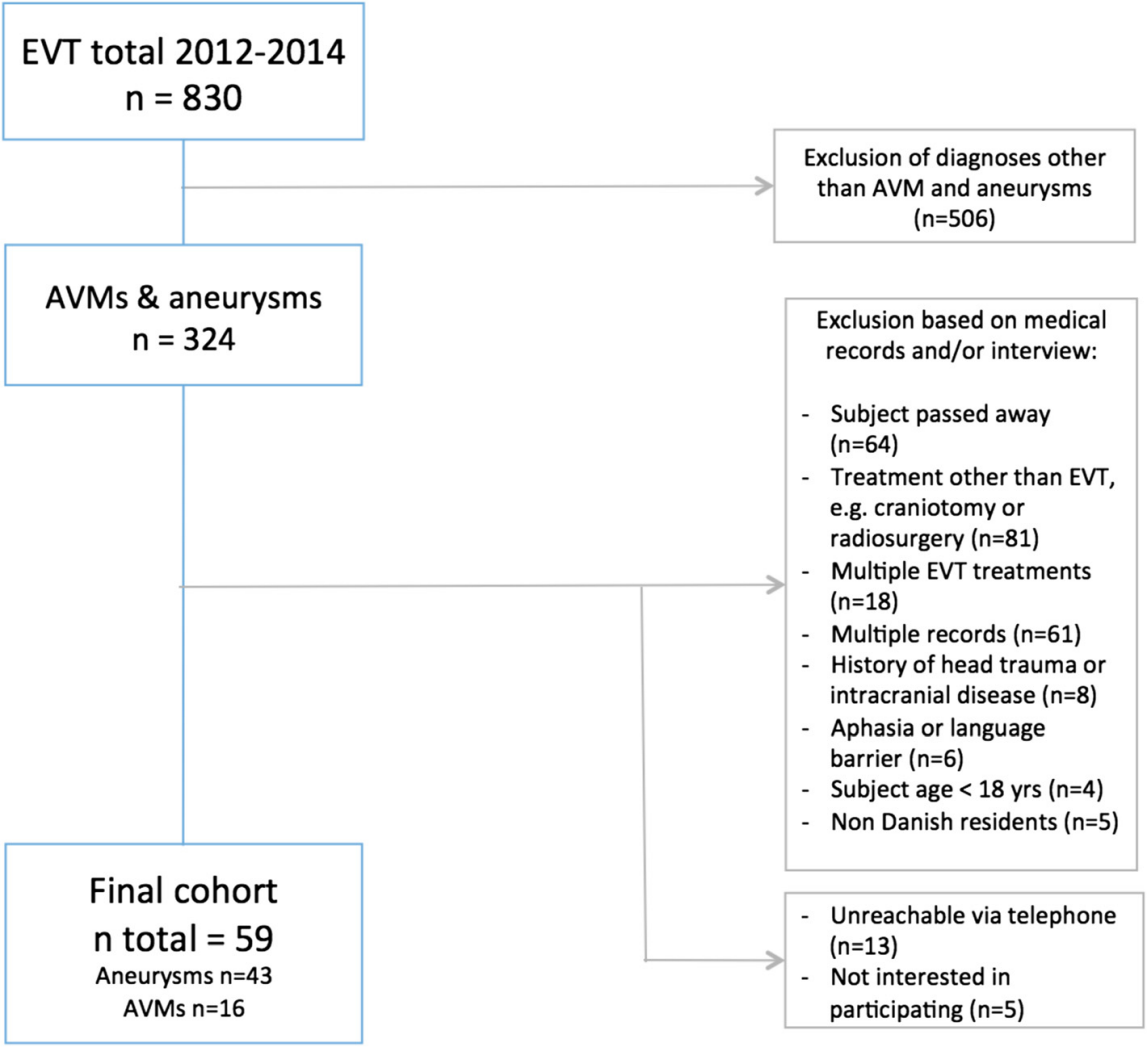
Study enrolment

A total of 830 patients were identified in the register. Of these patients, 324 had undergone EVT for aneurysms (*n* = 239) or AVMs (*n*= 85) (Fig. 1). The AVM group comprised patients that had undergone embolization for various malformations including arteriovenous fistulas, spinal dural arteriovenous fistulas, and dural arteriovenous fistulas (DAVFs). After application of exclusion criteria, a total of 59 eligible subjects were included in the study (43 treated for aneurysms, 16 treated for AVMs). Median age at time of EVT was 57 years (range: 30–87 years). Median time from EVT procedure to interview was 2.5 years (range: 1.2–3.8 years). Clinical and demographic characteristics of study participants are outlined in Table 1.

**Table 1:** Clinical and demographic characteristics of endovascularly treated patients

Characteristics	
Median age and range	57,4 (30–87)
Males	21
Right-handed	50
Median height and range (cm)	169 (154 – 203)
Median weight and range (kg)	74 (48 – 106)
Median BMI and range (kg/m^2^)	25 (17–39)
Vascular disorder	
- Aneurysms	43
- AVMs	5
- DAVFs	11
Aneurysm procedures	
- Coiling	26
- Stenting	11
- Coiling + stenting	4
- Web device	2
AVM procedures	
- Embolization	5
DAVF procedures	
- Embolization	9
- Embolization + coiling	2

AVM arteriovenous malformation, DAVF dural arteriovenous fistula

Patients treated for aneurysms underwent coiling, stenting, coiling and stenting combined, and placement of web-devices (table 1). Fifteen of the aneurysms had ruptured, 28 were unruptured. Patients treated with aneurysm coiling (*n* = 26) received standard antithrombotic care after EVT, which in Denmark consists of aspirin treatment for 2 months. Patients where aneurysms were treated with stent placement (*n* = 11), were prescribed aspirin for 6 months along with an additional antiplatelet medication for the first 3 months.

**Fig. 2:**
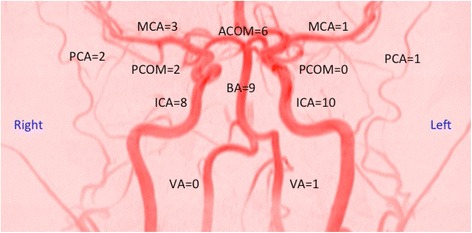
Localization of treated aneurysms, arteriovenous malformations, and dural arteriovenous fistulas. VA = vertebral artery, BA = basilar artery, ICA = internal carotid artery, MCA = middle cerebral artery, ACOM = anterior communicating artery, PCOM = posterior communicating artery, PCA = posterior cerebral artery. Another 16 localizations were distributed as follows: Left MCA + ICA = 1; bilateral ICA = 1; ophthalmic artery = 1; cavernous sinus = 1; left sigmoid sinus = 1, and unspecified locations (n = 11)

AVMs were treated with embolization solely (2 partial occlusions, 3 complete occlusions), while DAVFs were treated with embolization or embolization and coiling combined (2 partial occlusions, 9 complete occlusions). The most common localizations of vascular malformations, fistulas, and aneurysms were the internal carotid arteries and the basilar artery (Fig. 2).

### Headache status before and after EVT

**Fig. 3:**
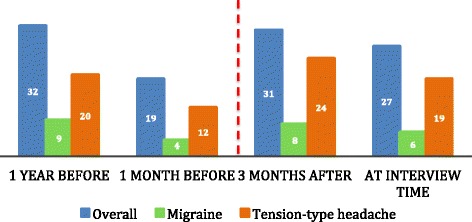
Headache status before and after endovascular treatment. Numbers represent patients. Overall headache includes migraine with aura, migraine without aura, tension-type headache, probable migraine, probable tension-type headache, cluster headache, and unclassified headache. Red dotted line represents the endovascular procedure

A similar number of patients experienced overall headaches 3 months after EVT compared with 1 year before the procedure (31 versus 32 patients; 23 treated for aneurysms, 8 treated for AVMs) (Fig. 3, Table 2). All but one patient with migraine continued to experience migraine attacks post-EVT (9 versus 8 subjects). Slightly more subjects experienced TTH 3 months after EVT compared with the year before the procedure (20 versus 24 subjects, *p* = 0.5). However, none of the above comparisons were statistically significant. Extending the post-EVT window of comparison from 3 months to “time of interview” (on average 2.5 years after EVT) had little impact on TTH (20 versus 19 subjects), while for migraine headaches; a larger albeit not statistically significant reduction was observed (9 versus 6 subjects, *p* = 0.5).

**Table 2:** Comparison of 59 patients’ headache status before and after endovascular treatment

	Anytime before (n)	Interview time (n)^a^		*P*-value
Overall headache	45	27		0.0003*
Migraine	15	6		0.022*
Tension-type headache	27	19		0.077
	1 year before (n)	Interview time (n)	Change (%)	*P*-value
Overall headache	32	27	−16	0.302
Migraine	9	6	−33	0.453
Tension-type headache	20	19	−5	1.000
	1 month before (n)	3 months after (n)	Change (%)	*P*-value
Overall headache	19	31	+63	0.017*
Migraine	4	8	+100	0.289
Tension-type headache	12	24	+100	0.012*

^**a**^Median time from EVT procedure to interview: 2.5 years

*P-values under 0.05

We also compared patients’ headache status 1 month before EVT with 3 months after EVT, as an expression of peri-procedural headache status. Here we found an increase in overall headache (19 versus 31 subjects, *p* = 0.017), tension-type headache (12 versus 24 subjects, *p* = 0.012), and migraine (4 versus 8 subjects, *p* = 0.3) within the first 3 months after EVT. At interview time, there was still an increase in overall headache (19 versus 27 subjects, *p* = 0.1; 19 treated for aneurysms, 8 treated for AVMs), migraine (4 versus 6 subjects, *p* = 0.7) and tension-type headache (12 versus 19 subjects, *p* = 0.1), though none of these results reached statistical significance.

We also asked patients about headaches experienced at any time prior to EVT, as a measure of pre-intervention lifetime headache prevalence in this patient group. Compared to headache experienced at interview time, there was a significant decrease in overall headache (45 versus 27 subjects, *p* < 0.001) and migraine (15 versus 6 subjects, *p* = 0.022).

In patients treated for aneurysms and who reported headache after EVT, coiling was the most prevalent procedure followed by stenting, both at 3 months after EVT (23 subjects with overall headache; coiling = 13, stent = 7) and at time of interview (19 subjects with overall headache; coiling = 11, stent = 5, coiling + stent = 12).

### Headache frequency before and after EVT

Comparing migraine frequency before and after EVT, there was an increase in headache days during the first 3 months post-procedure (31–45 headache days per 3 months) compared to both 1 year, and 1 month before EVT (6–12 headache days per month) (Table 3). However, at interview time, the frequency had regressed towards 1–5 headache days per month.

**Table 3:** Headache frequency before and after endovascular treatment

	Headache day score in relation to endovascular procedure			
	1 year before	1 month before	3 months after	At interview time
Migraine	2 (1 – 7) *n* = 9	2.5 (2 – 3) *n* = 4	5 (1 – 7) *n* = 8	1 (1 – 4) *n* = 6
Tension-type headache	3 (1 – 7) *n* = 20	1 (1 – 3) *n* = 12	2 (0 – 7) *n* = 24	1 (0 – 4) *n* = 19

Frequency represented as a headache score corresponding to following intervals: 0 = no headache

days, 1 = 1–5 headache days, 2 = 6–12 headache days, 3 = 13–24 headache days, 4 = 25–30 headache days, 5 = 31–45 headache days, 6 = 46–60 headache days, and 7 = more than 60 headache days. Headache scores listed as median and range

Headache frequency of TTH showed a similar pattern over time, although far less pronounced, with an increase in headache days the first 3 months after EVT (6–12 headache days per 3 months) and a regression to pre-EVT frequency at time of interview (1–5 headache days per month).

### De novo headaches after EVT

**Fig. 4:**
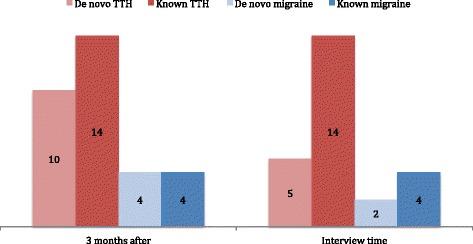
Distribution of de novo headaches and known headache after endovascular treatment. Numbers represent patients. TTH = tension-type headache

In all, 10 of 24 subjects with TTH post-procedure experienced this headache type for the first time in their lives during the first 3 months after EVT (Fig. 4). By the time of interview, only 5 patients still experienced these headaches.

First time ever attacks of migraine were experienced by 4 subjects in the 3 month period after EVT, and two of these patients still experienced migraine attacks at time of interview (2.1 and 2.6 years after EVT, respectively), with a median frequency of 25–45 headache days per 3 months.

There was no correlation between side of procedure and headache laterality (data not shown).

## Discussion

Existing studies on post-EVT headache in patients with aneurysms and AVMs show diverging results, generally favoring improvement of headache after treatment [8, 9]. Where an exacerbation was found, it was typically reported to be temporary and to resolve within days or months [6–8, 10]. Nevertheless, however short-lived these headaches may be, the majority still defy the strict time frame defined by the International Headache Classification, stating that headaches attributed to intracranial endovascular procedures develop within seconds and resolve within 24 h of the procedure [2]. For this reason, it has been suggested that ICHD-III beta criteria may not properly reflect clinical reality in terms of the temporal aspect of a possible headache after EVT [11]. Our data support this notion, as we found an increase in headache prevalence within the first three months after EVT compared to one month before EVT. At time of interview, this increase had regressed towards prevalence estimates similar to those reported in studies of the general Danish population for subjects with migraine. For tension-type headache, the prevalence also decreased over time, but was generally lower than corresponding numbers from Danish population-studies.

Possible reasons for these temporary headaches after EVT include local inflammation due to placement of foreign objects such as stents, coils or glue, and mechanical stimulation of the arterial wall [6, 8, 10]. Other explanations include changes in flow dynamics after EVT causing the release or stress of a venous overload [5, 11], possibly leading to post-procedural headache. This mechanism may be particularly true of AVMs.

A sub-group analysis of our cohort suggests that post-EVT headache may be more prevalent after coiling of aneurysms, followed by stent-placements. This is in line with previous studies, suggesting that use of a stent device is associated with increase of headache [6, 8]. It also seems that post-procedure headache is more prevalent in patients treated for aneurysms compared to AVMs. However, aneurysms are overrepresented in our cohort, and for this reason, along with generally small numbers in this subgroup analysis, we acknowledge that no firm conclusions can be made based on our tentative findings.

A potential correlation between aneurysms, AVMs and headache is no new concept, and studies have suggested that for patients harboring these vascular anomalies, headache may be more prevalent than in the general population [3, 4, 12]. Based on epidemiological studies of the general Danish population, the lifetime cumulated prevalence for both sexes is 16–18 % for migraine and 78 % for tension-type headache [13, 14]. With this study, we report a lifetime prevalence of 25 % for migraine, and 46 % for tension-type headache, suggesting that patients with aneurysms and AVMs are predisposed specifically to migraine. The pathophysiological mechanism responsible for this increase in migraine prevalence may be related to the very angioarchitecture of these vascular conditions, which, per definition, is abnormal. Lebedeva et al. suggested that increased sensory input from sensory nerve endings around the aneurysms may sensitize the central nervous system decreasing the threshold for developing spontaneous migraine attacks [4]. Other possible mechanisms predisposing to headache include local thrombosis, meningeal inflammation, expansion and inflammation of the aneurysm, and bleeding within the vessel wall; ultimately activating nociceptive C-fibers of the trigeminal nerve [4, 15]. For AVMs, Galletti et al. showed the location of AVMs to be significantly associated with migraine-like headache presentation, leading the authors to suggest that hemodynamic and structural changes caused by AVMs in the occipital lobe may trigger spreading depression and thus migraine [5].

For 1-year prevalence of headache, our data support the general knowledge of migraine prevalence, with 15 % reporting migraine in our cohort (9 out of 59 subjects within the last year prior to EVT) versus 10 % in the general Danish population [14]. The 1-year prevalence of tension-type headache is smaller in our cohort, with a prevalence of 34 % versus 74 % in the general Danish population [14]. In contrast to our results, Lebedeva et al. found a significant increase in the 1-year prevalence of migraine without aura in subjects with aneurysms (42.2 %) compared to controls (8.8 %), but no significant change in tension-type headache (19.6 % versus 23.1 %) [4]. However, important methodological differences exist between the studies, which impede direct comparisons. Subjects in the study by Lebedeva et al. were interviewed at time of admission to hospital upon rupture of their aneurysm. As thunderclap headache is the common presentation of aneurysms, the painful condition of the subjects at time of interview may have biased their recall of headaches. Conversely, in our study we interviewed patients after EVT, and it can be argued that the overall success of the procedure as seen from a patient perspective, may have influenced the subjects’ recollection of their headaches, e.g., with a successful procedure leading to less focus on or recall of concomitant headache. It can be argued that the inclusion of 15 patients with ruptured aneurysms at the time of EVT could influence our results in so far that patients with this condition can experience headaches up to 1 month after the event [2]. However, in a post hoc analysis we excluded these 15 patients, and found similar results (1 month before vs. 3 months after, migraine: 3 subjects vs 6 subjects; TTH: 7 subjects vs 19 subjects).

In our cohort, a fraction of patients experienced onset of new headache after EVT. Three months post EVT, 10 subjects experienced tension-type headache, and 4 subjects experienced migraine; these numbers declined to 5 and 2 subjects, respectively, at time of interview. Albeit small in numbers, these de novo headaches may still be indicative of a causal relationship between EVT and headache, especially when considering the cohort median age of 57 years. Migraine onset in this age group is highly unusual [16]. We also note that some patients experience only temporary headache after EVT while others progress to persistent headache. Schwedt and colleagues suggest that central sensitization may contribute to the persistence of any such new headache following EVT [8].

In terms of classification of these post-procedural headaches, any de novo headache occurring for the first time after EVT is a secondary headache and would optimally be classified as a “headache attributed to intracranial endovascular procedure” [2]. However, as per the current definition, this headache resolves within 24 h after EVT, thus not fitting the temporal pattern of headache observed in our cohort. By comparison, the classification for “acute headache attributed to craniotomy” allows for headache duration of up to 3 months after surgical intervention. We believe this time frame to be more appropriate for our results, which suggested an increased prevalence of headache for the first 3 months after EVT that thereafter regresses over time. Faced with these classification complexities, we chose to classify all post-procedural headaches as primary headaches, although we fully acknowledge that de novo headaches with a causal and temporal relation to EVT can be conceived as secondary headaches.

Strengths of this study include a subject group comprised of two vascular conditions both treated with the introduction of foreign bodies including coils, glue, and stenting. Subjects were consecutively included at a single tertiary referral center. All interviews were conducted by medical specialists trained in diagnosing headaches according to the ICHD-III beta, using purpose-developed questionnaires and through direct, telephone contact. Also, to describe the evolution of headaches over time, we specifically collected information on subjects’ headaches at multiple time-points before and after EVT. Finally, we pooled subjects with aneurysms and AVMs, as these conditions both comprise vascular anomalies and may predispose to headache by way of similar angioarchitectural mechanisms.

Because of its retrospective nature, recall bias is an inherent limitation of our study. Remembering characteristics of headaches occurring several years prior to EVT may pose a considerable challenge to some patients, which can result in some degree of headache diagnosis inaccuracy. Also, we recognize the limitation of our sample size. However, the relatively small sample size was partly due to a conscious choice on our behalf, i.e., our adherence to strict exclusion criteria so as to minimize bias introduced by inclusion of other conditions or treatments than the ones under study. Nonetheless, we cannot rule out the impact of clinical diversity on our results due to inter- and intra-group heterogeneities (e.g., aneurysms versus AVMs and a large aneurysm versus a small aneurysm, ruptured versus unruptured aneurysms, etc.), which we could only address in a limited fashion, i.e., in analyses stratified by type of vascular malformation (aneurysm vs. AVM).

For headache after EVT, we lack information on the time period between 3 months after EVT and up to the time of the interview, which took place 2.5 years after EVT on average. This would have provided us with a more detailed understanding of the evolution of a post-procedural headache.

Finally, one could argue that headache in the wake of such serious and potentially debilitating conditions is only a minor concern. Nevertheless, we do find it important to investigate functional outcomes after EVT in this patient group, particularly for those patients who are successfully treated for their primary condition, but where EVT results in other negative consequences such as persistent headache, which indeed may be highly disabling for the affected individual.

## Conclusion

In conclusion, our results indicate that EVT performed in subjects with intracranial aneurysms and AVMs leads to a temporary increase in headache for the first three months after EVT. Over time, this increase in headache status regresses towards prevalence estimates resembling those of the general Danish population. Also, new onset of headache in a smaller fraction of subjects experienced in the first three months after EVT resolves over time in half of the cases. This knowledge of a potentially short-lived headache after EVT holds significant clinical value, as it may lead to better counseling of subjects about to undergo EVT and optimization of treatment efforts in cases where post-procedural headache does occur. Finally, we tentatively suggest that the current ICHD-3 beta definition of headache attributed to intracranial vascular procedures may be sub-optimal, as our results show that any such headache may well last more than the allowed 24 h. Prospective studies are warranted to closely follow headache patterns within shorter time intervals after EVT. This will allow us to further uncover even small exacerbations in headache, which inadvertently may affect the quality of life for the individual patient, however temporary these changes may prove to be.
